# Comparison of conservative management, microsurgery only, and microsurgery with preoperative embolization for unruptured arteriovenous malformations: A propensity score weighted prospective cohort study

**DOI:** 10.1111/cns.14533

**Published:** 2023-11-21

**Authors:** Heze Han, Yu Chen, Li Ma, Ruinan Li, Zhipeng Li, Haibin Zhang, Kexin Yuan, Ke Wang, Hengwei Jin, Xiangyu Meng, Debin Yan, Yang Zhao, Yukun Zhang, Weitao Jin, Runting Li, Fa Lin, Qiang Hao, Hao Wang, Xun Ye, Shuai Kang, Dezhi Gao, Shibin Sun, Ali Liu, Youxiang Li, Xiaolin Chen, Yuanli Zhao, Shuo Wang

**Affiliations:** ^1^ Department of Neurosurgery, Beijing Tiantan Hospital Capital Medical University Beijing China; ^2^ China National Clinical Research Center for Neurological Diseases Beijing China; ^3^ Department of Interventional Neuroradiology, Beijing Tiantan Hospital Capital Medical University Beijing China; ^4^ Department of Neurosurgery, The First Hospital of Hebei Medical University Hebei Medical University Shijiazhuang China; ^5^ Department of Neurosurgery Shanxi Provincial People's Hospital Taiyuan Shanxi China; ^6^ Department of Neurosurgery, Peking University International Hospital Peking University Beijing China; ^7^ Department of Gamma‐Knife Center, Beijing Tiantan Hospital Capital Medical University Beijing China

**Keywords:** arteriovenous malformation, embolization, hemorrhagic stroke, microsurgery, neurologic deficit

## Abstract

**Aims:**

To compare the efficacy and deficiency of conservative management (CM), microsurgery (MS) only, and microsurgery with preoperative embolization (E + MS) for unruptured arteriovenous malformations (AVMs).

**Methods:**

We prospectively included unruptured AVMs undergoing CM, MS, and E + MS from our institution between August 2011 and August 2021. The primary outcomes were long‐term neurofunctional outcomes and hemorrhagic stroke and death. In addition to the comparisons among CM, MS, and E + MS, E + MS was divided into single‐staged hybrid and multi‐staged E + MS for further analysis. Stabilized inverse probability of treatment weighting using propensity scores was applied to control for confounders by treatment indication across the three groups.

**Results:**

Of 3758 consecutive AVMs admitted, 718 patients were included finally (266 CM, 364 MS, and 88 E + MS). The median follow‐up duration was 5.4 years. Compared with CM, interventions (MS and E + MS) were associated with neurological deterioration. MS could lower the risk of hemorrhagic stroke and death. Multi‐staged E + MS was associated with neurological deterioration and higher hemorrhagic risks compared with MS, but the hybrid E + MS operation significantly reduced the hemorrhage risk.

**Conclusion:**

In this study, unruptured AVMs receiving CM would expect better neurofunctional outcomes but bear higher risks of hemorrhage than MS or E + MS. The single‐staged hybrid E + MS might be promising in reducing inter‐procedural and subsequent hemorrhage.

## Graphical abstract

Microsurgery (MS) only and microsurgery with preoperative embolization (E + MS) could significantly prevent the hemorrhagic risk but caused more neurofunctional deficit than conservative management (CM).

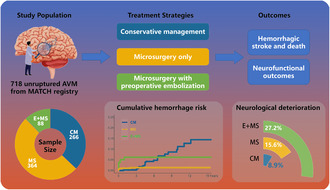

## 1 INTRODUCTION

Cerebral arteriovenous malformations (AVMs) are tangles of abnormally dilated vessels with the nidus fed by arteries and drained by veins without intervening capillaries.^1^ Hemorrhage is recognized as the primary adverse event if the lesion is left untreated.^2^ Although the ARUBA trial (A Randomized Trial of Unruptured Brain Arteriovenous Malformations) suggested that conservative management (CM) was superior to intervention in unruptured AVMs, several weaknesses rendered the conclusions underpowered.^3^, ^4^, ^5^ That said, the balance between the estimated cumulative lifetime hemorrhagic risk and the perceived postoperative complications must be weighed while selecting the therapeutic strategy.

Microsurgical resection remains a mainstay in the interventional treatment of AVMs, with higher rates of complete obliteration and immediate elimination of hemorrhage risk.^6^, ^7^ Adjunctive embolization before microsurgery was also applied with the initial purpose of facilitating the resection.^8^ However, whether to perform microsurgical resections and whether to embolize before microsurgery in unruptured AVMs remains debatable.^7^, ^8^, ^9^ Most studies investigating the benefit profile of microsurgery did not distinguish preoperative embolization from resection alone though the adjunctive procedure carried extra risks.^9^, ^10^, ^11^, ^12^ And studies evaluating beneficial effects of embolization to surgery typically mixed unruptured AVMs with those ruptured.^13^, ^14^, ^15^ These study designs with uncontrolled critical confounders would hamper the robustness and generalizability of their findings.

In this prospective study, we compared the neurofunctional outcomes and a composite endpoint of symptomatic hemorrhagic stroke and death on therapies among unruptured AVMs received CM, microsurgery (MS) only, and microsurgery with preoperative embolization (E + MS). Furthermore, we evaluated the different benefit profiles between the single‐staged hybrid operation and multi‐staged operation.

## 2 METHODS

### 2.1 Data source and the study design

This study was a retrospective cohort study aimed to compare the efficacy and deficiency of CM, MS, and E + MS for unruptured AVMs based on a prospectively acquired single‐center database recruited in the MATCH registry (Beijing Tiantan Hospital, the registry sponsor). The registry of multimodality treatment for brain AVMs in mainland China (MATCH study) was a nationwide multicenter prospective collaboration (ClinicalTials.gov register, NCT 04572568). This registry aimed to investigate the natural history of AVMs in the Asian population and explore the optimal therapeutic strategy for patients with AVMs.^16^ Previous publication have proved the validity of the registry.^17^ A detailed description of data quality management is shown in Method 1 in Data S1.

Patients with AVMs were continuously recruited in our institution from August 2011 to August 2021. Those with unruptured AVMs undergoing CM, MS, and E + MS were eligible for the study. The exclusion criteria were as follows: (1) patients missing critical clinical baseline data, pre‐treatment imaging, and lost to follow‐up; (2) ruptured AVMs at admission or before treatment; (3) patients undergoing stereotactic radiosurgery or stand‐alone embolization; (4) AVMs of Spetzler‐Martin (SM) grade V, as interventional treatment was not recommended in these patients at our institution, and the inclusion of them would violate the positivity assumption of the propensity score methods.^18^ The entire sample with follow‐up was included in the cohort analyses. The Institutional Review Board of Beijing Tiantan Hospital approved this study (IRB approval number: KY 2020‐003‐01), and patients who participated in the registry have granted written informed consent at admission. This study was reported in accordance with the STROBE guidelines for observational cohort studies.^19^

### 2.2 Baseline characteristics

This study recorded the demographic information (age and sex), clinical presentations (seizure, headache, neurological deficiency, and modified Rankin Scale [mRS] at admission), and radiographic features describing AVMs (location, size, and angiographic characteristics of feeding arteries, the nidus, and drainage veins). The radiological information was determined via digital subtraction angiography (DSA) and magnetic resonance imaging (MRI), and the definition of these features was in accordance with the reporting terminology guidelines.^20^ The angio‐architectural parameters were collected using pre‐interventional imaging and confirmed by neurosurgery residents well‐trained by credentialed senior neuroradiologists.

### 2.3 Treatment and outcomes

We divided the unruptured AVMs into three groups based on the therapeutic strategy after admitting to our institution: CM, MS, and E + MS. The preoperative embolization included the single‐staged hybrid operation and the multi‐staged operation, and the final decision was made after careful evaluation of the treatment strategies by neurosurgeons and interventional neuroradiologists and obtaining informed consent from patients. The single‐staged operation was performed in a hybrid angio‐surgical suite consisting of a surgical microscope and a flat biplane panel of DSA unit. The resections were performed by neurosurgeons with more than 15 years experience in the cerebrovascular field.

The primary outcomes of the study were the long‐term neurofunctional outcomes (disabling neurological deficit and neurological deterioration) and a composite endpoint of symptomatic hemorrhagic stroke and death. The disabling neurological deficit referred to a mRS > 2 at the last follow‐up. Neurological deterioration was defined as worsening mRS at follow‐up compared with the baseline condition. Symptomatic hemorrhagic stroke was the clinically symptomatic event (any new focal neurological deficit, seizure, or new‐onset dramatic headache) confirmed by imaging findings (intracranial hematoma or subarachnoid hemorrhage that could be attributed to AVM on computed tomography or MRI). The secondary outcomes included increased frequency of seizures (including de novo epilepsy), new‐onset transient neurological deficit, and new‐onset permanent neurological deficit. Changes in the frequency of seizures were evaluated subjectively by patients at follow‐up. The new‐onset neurological deficiency was assessed only in the two interventional groups as we aimed to compare the risk of postoperative complications between the two strategies. Seizures were not included in the analysis of neurological deficits.

Outcomes were assessed via phone interviews or record review by well‐trained clinical research coordinators at 3 months, annually (1, 2, and 3 years), and every 5 years after the treatment decision. The inception point of the follow‐up was the date of clinical presentation onset that led to the diagnosis of the AVM for CM, and the date of first surgery or preoperative embolization for the two interventional groups. The endpoint was the last follow‐up for neurofunctional outcomes and hemorrhage‐free survivors, or the date of symptomatic hemorrhagic stroke/death.

### 2.4 Controlling for confounders

We use propensity scores to control for pretreatment imbalances on baseline characteristics. Those factors that could potentially affect both the selection of particular therapeutic strategies and outcomes were specifically referred to as “confounding by indication”.^21^ Propensity score methods were recommended to address this issue in clinical research.^22^ In this study, we applied stabilized inverse probability of treatment weighting (sIPTW) using generalized boosting models to generate stabilized weights across the three treatment groups.^23^ All available covariates that were included in the generalized boosting model. This method was developed for comparing multiple treatments and has been widely validated in previous studies.^24^, ^25^ These stabilized weights allowed us to estimate the average treatment effect with no patients excluded and the original sample size similarly preserved.^26^ The balance diagnostic tests were assessed using standardized mean differences (SMD) before and after weighting. An SMD of 0.2 or less was regarded as an acceptable balance.^27^ Despite the application of sIPTW, unmeasured confounders could still result in the failure of eliminating this form of bias. Therefore, *E*‐values were calculated for investigation of the strength of unmeasured confounders needed to explain away the observed association.^28^

### 2.5 Statistical analyses

We used R software (version 4.0.3) to perform statistical analyses. Statistical significance was set at two‐sided *p* < 0.05. Baseline characteristics with SMD before and after sIPTW were tabulated for each treatment group. Continuous variables were reported as mean ± SD or median (interquartile ranges [IQR]) for normal and non‐normal distribution data. The proportion (*n*%) of each categorical variable was also recorded. Kaplan–Meier curves were plotted for different treatment cohorts to visualize the incidence of symptomatic hemorrhagic stroke and death. All the subsequent analyses were conducted using sIPTW, except for the crude and the regression adjustment analyses. The odds ratios (ORs) were calculated using logistic regression model for neurofunctional outcomes and all the secondary outcomes. Hazard ratios (HRs) were estimated using the Cox proportional hazards regression model for symptomatic hemorrhagic stroke and death. The proportional hazards assumption was assessed by examining Schoenfeld's global test, and HRs would be reported at 3, 5, and 10 years if the assumption was violated.^29^

The overall data analysis protocol was as follows. Stage 1: Comparisons were made between CM and interventional treatment (CM vs. MS and CM vs. E + MS); Stage 2: The two surgical therapies were then compared to evaluate the efficacy of preoperative embolization (MS vs. E + MS); Stage 3: In the E + MS arm, strategies were further divided into hybrid operation and multi‐staged operation to investigate whether the single‐staged operation was superior to multi‐staged procedures.

In sensitivity analyses, we compared the effect of the two interventions with CM on the primary outcomes by applying different statistical methods and including selected patients. The crude analysis without weighting or adjusting was conducted. We also performed the multivariable logistic regression and Cox‐proportional hazard model adjusting for factors included in the propensity score calculation to estimate the ORs and HRs of study outcomes. To compare our results with the ARUBA study, we further included only ARUBA‐eligible patients. Finally, to distinguish whether single‐staged hybrid operation and multi‐staged preoperative embolization played different roles in the outcomes, patients undergoing these two procedures were separately included to form the preoperative embolization cohort as two independent sensitivity analyses.

Prespecified subgroup analyses stratified with respect to SM grades (I–II vs. III–IV), size (<3 cm vs. ≥3 cm), and eloquence regions (yes vs. no) were conducted in the neurofunctional outcomes, and the former two subgroups in the composite outcome. CM was set as the reference in these comparisons. Interaction tests were also recorded for these subgroups to assess the across‐subgroup heterogeneity.

## 3 RESULTS

### 3.1 Patient characteristics

**FIGURE 1:**
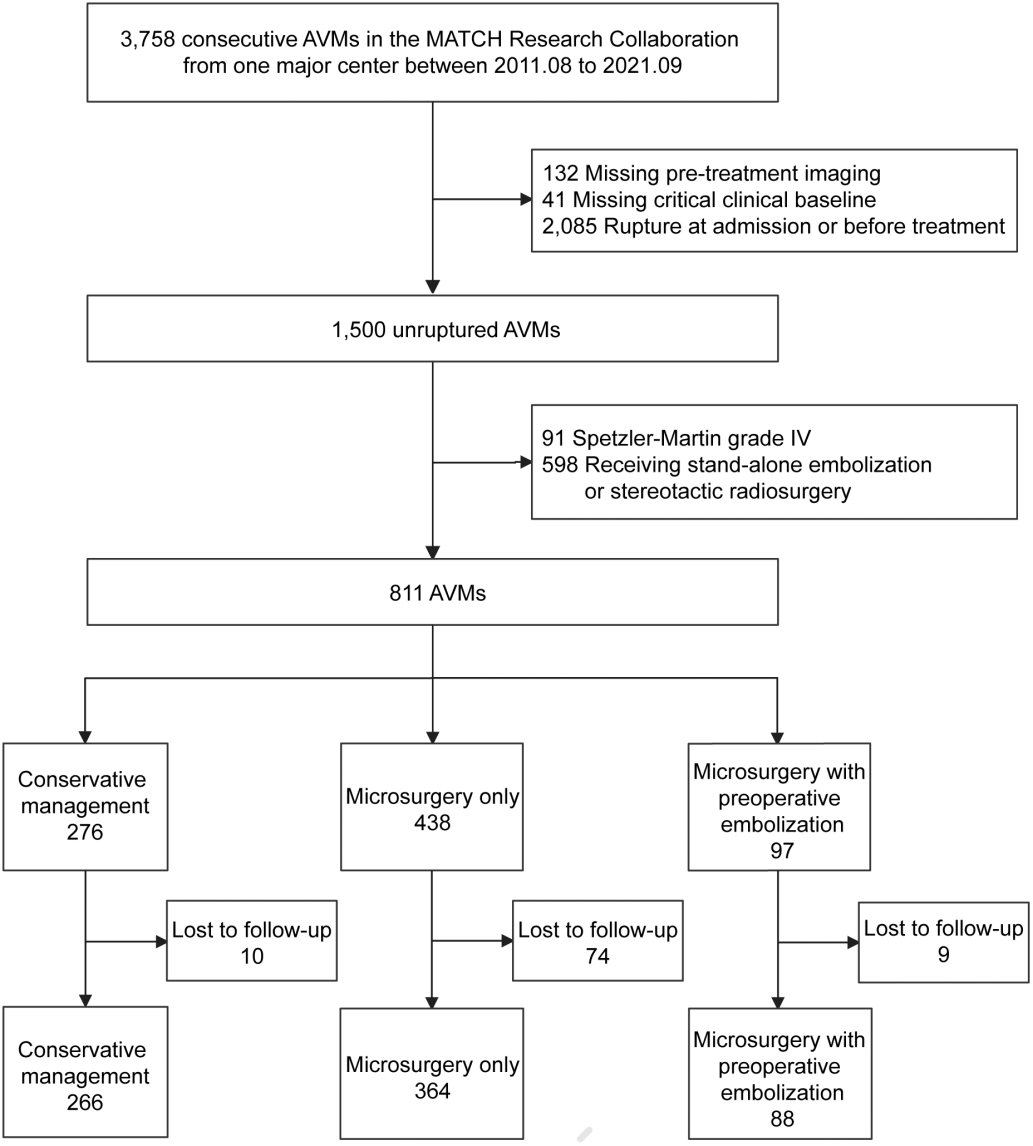
Flowchart of patient selection. AVM, arteriovenous malformation; MATCH, registry of multimodality treatment for brain AVMs in mainland China.

A total of 3758 patients diagnosed with AVMs were enrolled in the MATCH registry between August 2011 to August 2021 from our institution. Finally, we included 718 eligible unruptured AVMs with long‐term follow‐up data for further analysis. Among them, 266 (37.0%) received CM, 364 (50.7%) underwent MS, and 88 (12.3%) had E + MS. The median (IQR) follow‐up duration was 5.4 (3.2–8.2) years (CM: 5.9 [3.1–9.6] years; MS: 5.8 [3.3–8.1] years; E + MS: 4.5 [2.6–5.4] years). Figure 1 shows the details of how patients were selected. AVMs with perforating feeding arteries, diffuse nidus, exclusive deep drainage, eloquence location, and high SM grades were more likely to receive CM. And patients undergoing interventional treatment were prone to complain of seizures. Characteristics of patients lost to follow‐up were shown in Table S1. After sIPTW, baseline characteristics among the three treatment groups achieved an acceptable balance (Table 1, Figures S1 and S2).

**TABLE 1:** Baseline characteristics before and after stabilized inverse probability of treatment weighting.

	Before weighting				After weighting			
	Patients, No. (%)			SMD	Patients, No. (%)			SMD
	CM	MS	E + MS		CM	MS	E + MS	
Sample size	266	364	88		208	321	65	
Female	110 (41.4)	126 (34.6)	39 (44.3)	0.133	86 (41.4)	117 (36.5)	25 (39.4)	0.068
Age at diagnosis (median (IQR))	28.3 (17.3–39.2)	25.7 (17.0–36.2)	24.6 (15.9–32.0)	0.208	27.2 (16.8–36.7)	25.8 (17.0–36.3)	25.8 (16.4–34.1)	0.063
mRS at admission								
0	61 (22.9)	42 (11.5)	8 (9.1)	0.287	36 (17.4)	40 (12.6)	7 (10.1)	0.182
1	165 (62.0)	280 (76.9)	69 (78.4)		144 (69.4)	238 (74.2)	51 (77.8)	
2	35 (13.2)	36 (9.9)	10 (11.4)		24 (11.4)	35 (10.9)	7 (11.1)	
3	5 (1.9)	6 (1.6)	1 (1.1)		4 (1.8)	8 (2.4)	1 (1.0)	
Seizure	94 (35.3)	185 (50.8)	42 (47.7)	0.211	83 (39.9)	155 (48.2)	31 (47.1)	0.112
Headache	89 (33.5)	123 (33.8)	35 (39.8)	0.088	73 (35.1)	115 (36.0)	23 (35.2)	0.011
Neurological deficit	61 (22.9)	43 (11.8)	15 (17.0)	0.198	43 (20.8)	43 (13.3)	10 (14.9)	0.134
Location								
Frontal	87 (32.7)	144 (39.6)	34 (38.6)	0.095	73 (34.9)	119 (37.2)	26 (39.2)	0.059
Temporal	76 (28.6)	120 (33.0)	22 (25.0)	0.117	64 (30.6)	100 (31.1)	17 (26.4)	0.069
Parietal	106 (39.8)	76 (20.9)	23 (26.1)	0.280	70 (33.4)	85 (26.3)	19 (29.3)	0.102
Occipital	48 (18.0)	80 (22.0)	30 (34.1)	0.247	40 (19.2)	78 (24.2)	19 (29.7)	0.164
Cerebellum	26 (9.8)	15 (4.1)	4 (4.5)	0.149	22 (10.4)	14 (4.4)	3 (4.4)	0.155
Basal ganglia	20 (7.5)	4 (1.1)	1 (1.1)	0.214	10 (4.9)	5 (1.6)	1 (1.6)	0.126
Spetzler‐Martin grade								
1	24 (9.0)	72 (19.8)	9 (10.2)	0.424	21 (10.2)	52 (16.1)	8 (13.2)	0.212
2	71 (26.7)	147 (40.4)	32 (36.4)		70 (33.7)	116 (36.2)	24 (37.4)	
3	100 (37.6)	112 (30.8)	35 (39.8)		76 (36.4)	112 (34.8)	25 (39.0)	
4	71 (26.7)	33 (9.1)	12 (13.6)		41 (19.6)	42 (12.9)	7 (10.3)	
Ventricular system involvement	95 (35.7)	73 (20.1)	31 (35.2)	0.236	62 (29.9)	80 (25.0)	19 (29.5)	0.072
Size								
<3 cm	68 (25.6)	128 (35.2)	20 (22.7)	0.184	57 (27.2)	102 (31.7)	16 (24.3)	0.111
≥3 cm	198 (74.4)	236 (64.8)	68 (77.3)		152 (72.8)	219 (68.3)	49 (75.7)	
Eloquent region	155 (58.3)	142 (39.0)	39 (44.3)	0.261	108 (51.9)	143 (44.6)	28 (42.9)	0.120
Feeding artery dilation	177 (66.5)	230 (63.2)	67 (76.1)	0.189	133 (63.8)	207 (64.5)	48 (74.3)	0.153
Single feeder	33 (12.4)	65 (17.9)	17 (19.3)	0.127	31 (15.0)	53 (16.4)	11 (16.5)	0.028
Perforating artery	110 (41.4)	58 (15.9)	25 (28.4)	0.388	64 (30.8)	73 (22.9)	18 (27.0)	0.120
Aneurysm	47 (17.7)	52 (14.3)	11 (12.5)	0.097	33 (16.0)	47 (14.7)	11 (16.4)	0.032
Diffuse nidus	92 (34.6)	65 (17.9)	21 (23.9)	0.258	59 (28.2)	68 (21.3)	16 (24.6)	0.106
Any deep drainage	78 (29.3)	61 (16.8)	24 (27.3)	0.201	53 (25.5)	66 (20.5)	14 (20.8)	0.079
Draining vein stenosis	29 (10.9)	42 (11.5)	9 (10.2)	0.028	20 (9.7)	37 (11.6)	6 (9.5)	0.044
Venous aneurysm	84 (31.6)	101 (27.7)	27 (30.7)	0.056	58 (27.9)	91 (28.3)	20 (30.6)	0.039

*Abbreviations*: CM, conservative management; E + MS, microsurgery with preoperative embolization; IQR, interquartile range; mRS, modified Rankin scale; MS, microsurgery only; SMD, standardized mean differences.

### 3.2 CM versus interventional treatment

**FIGURE 2:**
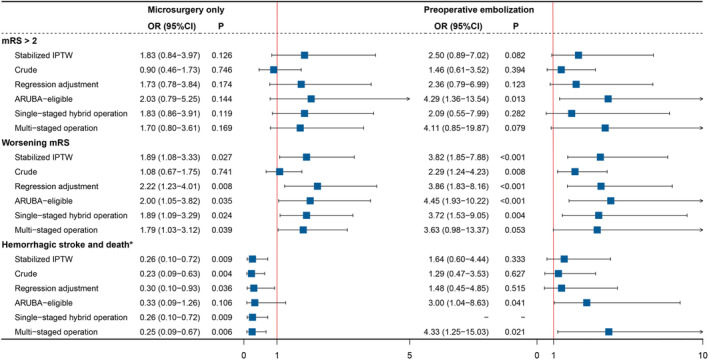
The effect size and sensitivity analysis for primary outcomes by therapeutic strategies. *The effect size of hemorrhagic stroke and death was expressed as hazard ratios. ARUBA, A Randomized Trial of Unruptured Brain Arteriovenous Malformations; CI, confidence interval; IPTW, inverse probability of treatment weighting; mRS, modified Rankin scale; OR, odds ratio.

The breakdown of the frequency of each outcome was shown in Table 2. Overall, both interventions resulted in poorer long‐term neurofunctional outcomes and lower risk of symptomatic hemorrhagic stroke/death than CM. After sIPTW, the three strategies did not show a significant difference in disabling outcomes (OR 1.83, 95% CI 0.84–3.97 for MS; OR 2.50, 95% CI 0.89–7.02 for E + MS, comparing with CM, Figure 2). However, both interventional treatments could lead to neurological deterioration at follow‐up (OR 1.89, 95% CI 1.08–3.33, *E*‐value 3.20 for MS; OR 3.82, 95% CI 1.85–7.88, *E*‐value 7.10 for E + MS, Figure 2).

**TABLE 2:** Outcomes of different therapeutic strategies after stabilized inverse probability of treatment weighting.

Outcomes	CM (*n* = 208)	MS (*n* = 321)			E + MS (*n* = 65)		
	Events (%)	Events (%)	Relative risk (95% CI)^a^	Attributable risk (95% CI)^a^	Events (%)	Relative risk (95% CI)^a^	Attributable risk (95% CI)^a^
Primary outcome							
mRS > 2	9.3 (4.5)	25.2 (7.9)	1.76 (0.85–3.66)	0.03 (−0.01–0.08)	6.8 (10.4)	2.34 (0.91–6.04)	0.06 (−0.01–0.12)
Worsening mRS	18.6 (8.9)	50.1 (15.6)	1.75 (1.06–2.89)^b^	0.07 (0.01–0.13)^b^	17.7 (27.2)	3.04 (1.69–5.48)^b^	0.18 (0.09–0.28)^b^
Hemorrhagic stroke and death	14.0 (6.7)	4.2 (1.3)	0.19 (0.07–0.57)^b^	−0.05 (−0.09 to −0.02)^b^	3.9 (6.0)	0.89 (0.30–2.64)	−0.01 (−0.08–0.06)
Symptomatic hemorrhagic stroke	8.7 (4.2)	2.5 (0.8)	0.19 (0.05–0.75)^b^	−0.03 (−0.06 to −0.01)^b^	2.9 (4.5)	1.07 (0.29–3.91)	0.00 (−0.05–0.06)
Death	5.2 (2.5)	1.7 (0.6)	0.21 (0.04–1.19)	0.02 (−0.04–0.00)	1.0 (1.5)	0.62 (0.07–5.14)	0.01 (−0.05–0.03)
Secondary outcome							
Increased frequency of seizures	10.1 (4.8)	5.9 (1.9)	0.38 (0.14–1.03)	−0.01 (−0.03–0.02)	2.2 (3.3)	0.70 (0.17–2.92)	0.01 (−0.04–0.05)
New‐onset transient ND	–	56.9 (17.7)	–	–	18.4 (28.2)	–	–
New‐onset permanent ND	–	11.9 (3.7)	–	–	5.8 (8.8)	–	–

*Abbreviations*: CM, conservative management; CI, confidence interval; E + MS, microsurgery with preoperative embolization; mRS, modified Rankin scale; MS, microsurgery only; ND, neurological deficit.

^a^ The relative risk and attributable risk were calculated with the CM group as the reference.

^b^ Values with statistical significance.

**FIGURE 3:**
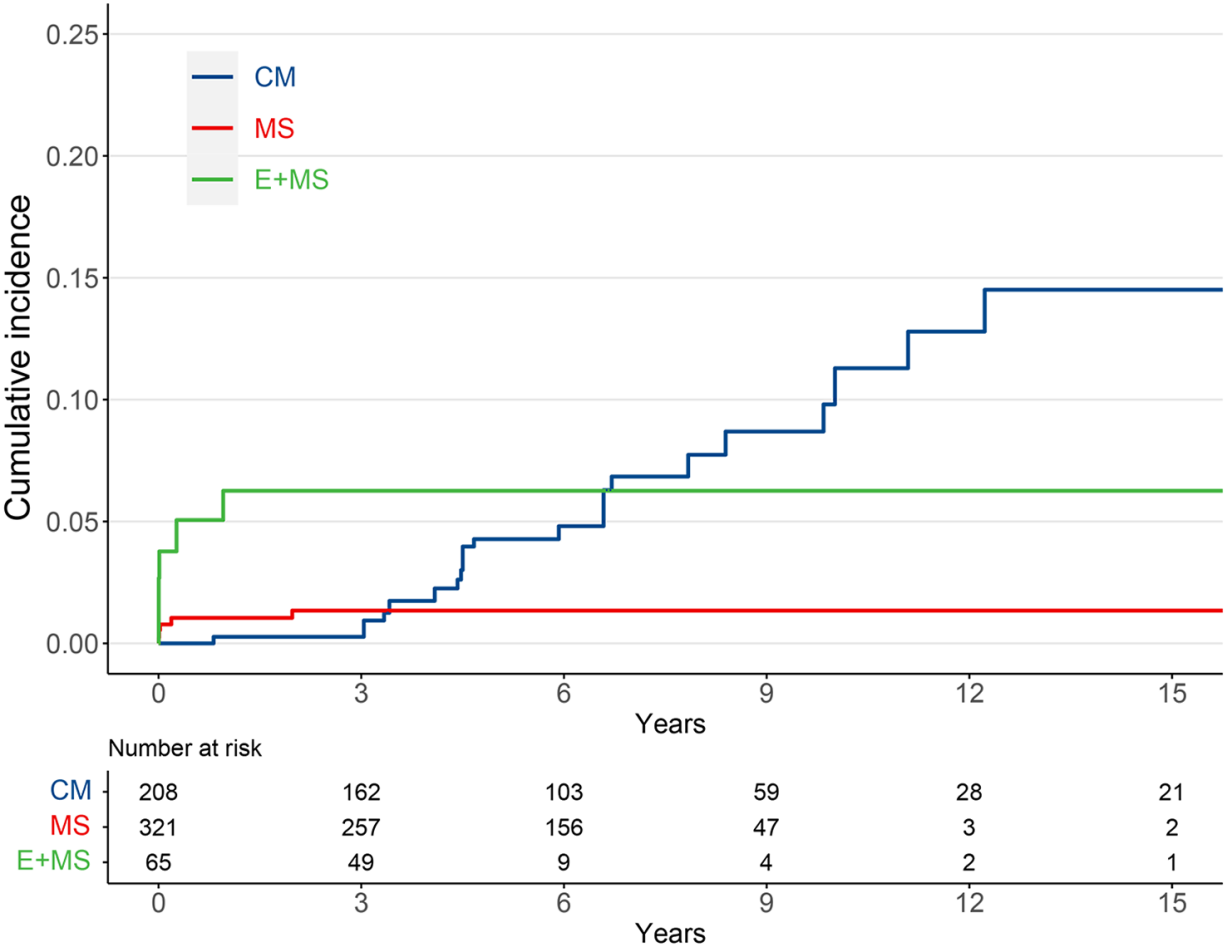
Weighted cumulative incidence for hemorrhagic stroke and death by therapeutic strategies. CM, conservative management; E + MS, microsurgery with preoperative embolization; MS, microsurgery only.

Interestingly, the MS observed lower cumulative hazards risk of symptomatic hemorrhagic stroke and death (HR 0.26, 95% CI 0.10–0.72, *E* value 7.20), while the E + MS shared a similar risk profile with CM (HR 1.64, 95% CI 0.60–4.44). Due to the violation of the proportional hazard assumption (*p* = 0.011), the follow‐up period was shortened to 3, 5, and 10 years to calculate the staged HRs (Table S2). The risk of symptomatic hemorrhagic stroke and death was higher than CM in the first 3 years both in the MS cohort (HR 5.58, 95% CI 0.62–50.29) and the E + MS cohort (HR 21.80, 95% CI 2.51–190.10). And the HRs decreased to insignificant (HR 1.51, 95% CI 0.53–4.38 for E + MS at 10‐year follow‐up) or even protective (HR 0.21, 95% CI 0.07–0.60 for MS at 10‐year follow‐up) as the observation duration increased. The weighted Kaplan–Meier plot also illustrated the crossovers at the 4th and the 7th years (Figure 3).

### 3.3 MS versus E + MS

In the comparison of MS versus E + MS, preoperative embolization was associated with worse neurofunctional outcomes (OR 2.02, 95% CI 1.08–3.77 for neurological deterioration, Table S3) and higher risks of symptomatic hemorrhagic stroke and death (HR 4.76, 95% CI 1.37–16.55, Table S3). When the E + MS was divided into hybrid operation (*n* = 63) and multi‐staged operation (*n* = 25) for further analyses, the differences against the MS cohort disappeared in terms of neurological deterioration (OR 1.97, 95% CI 0.96–4.03 for hybrid operation; OR 2.15, 95% CI 0.75–6.13 for multi‐staged operation; Table S4). However, the risk of symptomatic hemorrhagic stroke and death significantly increased after the multi‐staged operation (HR 18.13, 95% CI 5.22–62.92; Table S3), with all the hemorrhage occurring in the interval between embolization and microsurgery. A total of 32 embolization was conducted in 25 patients in the multi‐staged operation group, with the median (IQR) interval between the first embolization and surgical resection of 6.1 (4.2–17.3) months. The hybrid operation group observed no hemorrhagic event with a relatively short total follow‐up duration of 199 person‐years.

In terms of the postoperative complications, no higher risk in the E + MS cohort was observed overall compared with the MS cohort (increased frequency of seizures: OR 1.82, 95% CI 0.37–8.83; new‐onset transient ND: OR 1.82, 95% CI 0.99–3.36; new‐onset permanent ND: OR 2.53, 95% CI 0.90–7.10, Table S3). When analyzing the E + MS separately, the multi‐staged operation was more likely to carry a higher risk of long‐term permanent ND (OR 7.78, 95% CI 2.29–26.47, Table S4).

### 3.4 Sensitivity analyses

The sensitivity analyses (Figure 2) consistently revealed that patients undergoing interventional treatment were more likely to experience significantly higher risks of neurological deterioration. The results of the disabling outcomes comparing the interventional arms and CM were replicated by most sensitivity analyses with insignificant higher risk. The exceptions were the comparisons of ARUBA‐eligible patients in the E + MS cohort, in which the trends became significant (OR 4.29, 95% CI 1.36–13.54, *p* = 0.013). Similar inconsistency was observed in the symptomatic hemorrhagic stroke and death comparison of the MS cohort, as all other analyses indicated the MS significantly reduced the risk except for the ARUBA‐eligible method (HR 0.33, 95% CI 0.09–1.26, *p* = 0.106).

### 3.5 Subgroup analyses

**FIGURE 4:**
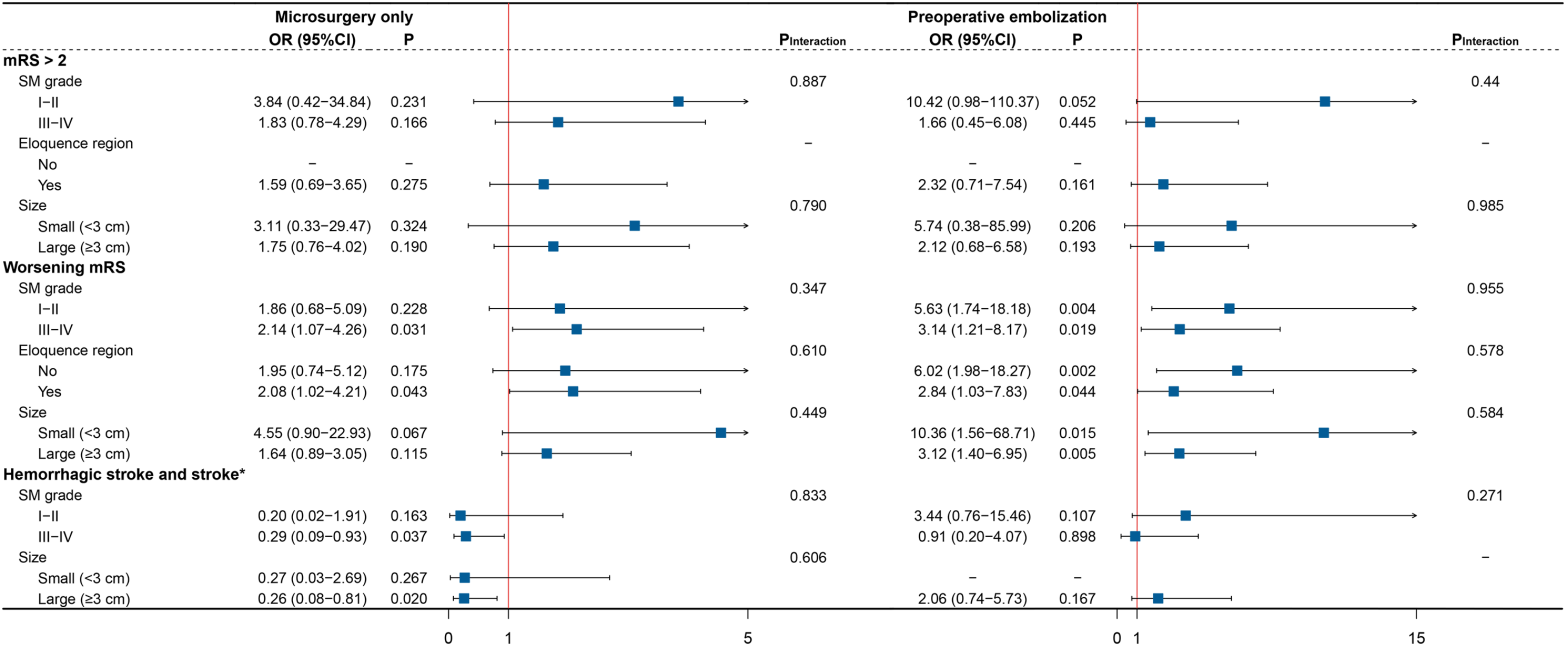
Subgroup analysis for primary outcomes. The effect size of hemorrhagic stroke and death was expressed as hazard ratios. CI, confidence interval; mRS, modified Rankin scale; OR, odds ratio; SM, Spetzler‐Martin.

Figure 4 summarized the results of the prespecified subgroup analyses. Results of the comparison of E + MS and CM were consistent across subgroups with no interaction effect. In the MS versus CM comparison, AVMs with higher SM grades (OR 2.14, 95% CI 1.07–4.26, *p* = 0.031) or eloquent location (OR 2.08, 95% CI 1.02–4.21, *p* = 0.043) were more likely to suffer from neurological deterioration. The subgroup results also suggest that in the E + MS versus CM comparison, SM I–II grade (OR 5.63, 95% CI 1.74–18.18, *p* = 0.004), non‐eloquent (OR 6.02, 95% CI 1.98–18.27, *p* = 0.002) or smaller AVMs (OR 10.36, 95% CI 1.56–68.71, *p* = 0.015) would bear higher risk of neurological deterioration than higher SM grades (OR 3.14, 95% CI 1.21–8.17, *p* = 0.019), eloquent‐seated (OR 2.84, 95% CI 1.03–7.83, *p* = 0.044), or larger AVMs (OR 3.12, 95% CI 1.40–6.95, *p* = 0.005), indicating the choice of preoperative embolization should be carefully considered in these less‐complicated AVMs. The subgroup analysis of long‐term hemorrhage and death indicated that patients with SM III–IV grade (HR 0.29, 95% CI 0.09–0.93, *p* = 0.037) or large‐size (HR 0.26, 95% CI 0.08–0.81, *p* = 0.020) AVMs might benefit more from surgical resection.

## 4 DISCUSSION

In this prospective cohort study, we compared different therapeutic strategies (CM, MS, and E + MS) for unruptured AVMs, and found that patients undergoing CM fared better than those with interventions in the neurofunctional outcomes, while the surgical resection was associated with lower risk of symptomatic hemorrhagic stroke and death. Compared with the single‐staged hybrid operation, the multi‐staged E + MS was linked with increased hemorrhage risk and permanent neurological deficit. These results contribute to the individualized evaluation between the risk of disability and hemorrhage according to treatment strategies.

In previous studies investigating the effect of surgical resection versus CM on unruptured AVMs, the preoperative embolization was typically not distinguished from MS alone.^10^, ^11^, ^12^, ^30^ However, as was indicated in the ARUBA trial, embolization carried a higher risk of stroke than MS. As such, the benefit profile of these strategies should be evaluated separately.^3^, ^5^ Studies assessing the additional benefits of adjunctive embolization before surgery were mostly conducted in a mixed population with about half of AVMs ruptured at initial presentation.^13^, ^14^, ^15^, ^31^ This could lead to potential bias as the hemorrhage was a protective factor to surgical resection in the supplementary grading scale, whether it resulted from spontaneous rupture or embolization.^32^ However, Andreas Hartmann et al. suggested that unruptured AVMs were less likely to benefit from the interventional treatment.^33^ In addition to the patient selection, a systematic review also criticized that most studies evaluating treatment effects failed to correct for underlying confounders.^8^ To the best of our knowledge, this is the first study to date comparing multiple treatment strategies in unruptured AVMs with sIPTW balancing the baseline characteristics among groups, as we aimed to provide comprehensive and robust evidence for more individualized recommendations in this post‐ARUBA era.^23^

This study demonstrated that patients receiving CM could experience less disability and neurological deterioration, which was in accordance with the neurofunctional impairment analysis of the ARUBA trial.^4^ However, in the sensitivity analysis of the ARUBA‐eligible patients in our research, such criteria could potentially exaggerate the disabling impact (E + MS vs. CM: OR 4.29, 95% CI 1.36–13.54, *p* = 0.013) and attenuate the beneficial effects of interventions in preventing symptomatic hemorrhagic stroke and death (MS vs. CM: OR 0.33, 95% CI 0.09–1.26), as they excluded younger patients that could benefit more from surgical resection.^11^ Consistent with previous literature, our results showed protective effects of MS on the hemorrhage occurrence with a rate far lower than that in the ARUBA trial.^10^, ^12^ In the E + MS group, all these events occurred in the interval between embolization and microsurgery in the multi‐staged operation cohort.

Previous studies evaluating the role of preoperative embolization focused on benefiting the surgery, which can be translated into reducing blood loss, shortening operative time, and increasing obliteration rates.^8^, ^31^, ^34^, ^35^ However, many researchers suggested contradicting findings, and they proposed that making surgery easier may not make the overall management safer.^36^ As such, we set the long‐term neurofunctional status and symptomatic hemorrhagic stroke or death as the primary outcomes and postoperative complications as the secondary outcomes to assess the practical role of the adjunctive procedure. Consensus has not yet been reached about whether the preoperative embolization improved the prognosis.^9^, ^13^, ^14^, ^15^, ^31^, ^37^ Catapano et al. found a decreased risk of poor neurological outcomes in SM III grade patients undergoing E + MS compared with MS.^14^ However, the follow‐up started immediately before resection. This measurement would omit the major adverse events that happened during the embolization and resection interval, which was reported to be a high incidence rate.^8^ Several studies have also suggested that the benefits of embolization should be weighed against the hemorrhage risk it carried.^31^, ^38^ As such, the single‐staged hybrid operation could be an option for safer outcomes by reducing the inter‐procedure interval.^39^ Our results supported this idea with no hemorrhagic events in the hybrid operation group and 3.61% per person‐year in the multi‐staged group, despite the short follow‐up duration (199 person‐years for hybrid operation and 108 for multi‐staged operation) that warranted cautious interpretation. It is expected that the parametric color‐coded angiography could serve as a hemodynamic analysis tool after embolization to assess whether the procedure actually reduces the hemorrhage risk.^35^, ^40^

Our study had several limitations. First, most AVMs in the E + MS group used Onyx as the liquid embolic agents and were partially embolized, so the effect of other agents and embolization strategies were not compared. However, previous studies have shown that preoperative embolization with Onyx did not differ from or even worked better than other embolic agents.^41^, ^42^ Future studies evaluating preoperative embolization should consider the embolization strategies as studies reported different risks of them.^43^ Second, the sample size in the E + MS group was limited compared with the other two groups, and the hybrid operation group observed no hemorrhagic event with a relatively short total follow‐up duration of 199 person‐years, so the comparison of the hemorrhagic events in this group should be interpreted with caution as the mobility was relatively low. And further studies with a larger sample size and longer follow‐up duration were required to confirm the result. Third, patients perceived risk of operative complications might shift from the MS strategy to the CM or be excluded for receiving palliative embolization without scheduled surgical resection, which could result in underestimating the adverse effect of interventional treatment. Such selection bias was inevitable in observational studies, and we have applied sIPTW to minimize the confounders by indication and enable the baseline characteristics to be comparable. Well‐designed randomized clinical trials are still needed to assess the benefit of interventional treatment for unruptured AVMs in the future. Fourth, despite the fact that the propensity score weighting was an excellent method to balance the observed baseline information while retaining the generalizability of the study population, the choice of treatment may not easily be ascribable to existing metrics. However, the *E*‐values of more than 3.00 meant that unmeasured confounders would have to be at a large magnitude to explain away our findings.

## 5 CONCLUSIONS

In this study, we found that unruptured AVMs receiving CM could expect better neurofunctional outcomes than those undergoing interventional treatment, while carrying a higher risk of symptomatic hemorrhagic stroke and death than MS or E + MS. In the comparison of E + MS and MS, the single‐staged hybrid operation, rather than multi‐staged E + MS, was recommended for reduced hemorrhagic events. Further subgroup analysis suggested a higher neurological deterioration and hemorrhage risk of E + MS in SM I‐II grade AVMs. Therapeutic decisions in patients with unruptured AVMs remain a complex task that requires multidisciplinary and individualized evaluation of the corresponding risks and benefits.

### ACKNOWLEDGEMENT

We thank all the staff for their contribution to this study and participant hospitals of the MATCH registry for their support.

## FUNDING INFORMATION

This work was supported by the National Key Research and Development Program of China (Grant No. 2021YFC2501101 and 2020YFC2004701 to Xiaolin Chen), the Natural Science Foundation of China (grant no. 81771234 and 82071302 to Yuanli Zhao, and 82202244 to Yu Chen).

## CONFLICT OF INTEREST STATEMENT

The authors declare that they have no conflicts of interest.

## Supporting information


Data S1.

## Data Availability

The data that support the findings of this study are available from the corresponding author upon reasonable request.
