# Supplementary material for: Comparison of conservative management, microsurgery only, and microsurgery with preoperative embolization for unruptured arteriovenous malformations: A propensity score weighted prospective cohort study
Source: CNS Neurosci Ther. 2023 Nov 21;30(4):e14533. doi: 10.1111/cns.14533 (PMC11017441; doi:10.1111/cns.14533)
Supplement: Supplementary file 1 — Data S1. [file CNS-30-e14533-s001.docx]

Content

[**Supplementary Method 1. Protocol for data quality management.** 2](#_Toc136030740)

[**Supplementary Figure 1. Overlap assessment** 4](#_Toc136030741)

[**Supplementary Figure 2. Effect size plots for assessing the balance of pretreatment variables** 5](#_Toc136030742)

[**Supplementary Table 2.** **Hazard ratios of different therapeutic strategies after 3, 5, and 10 years for symptomatic hemorrhagic stroke and death.** 6](#_Toc136030743)

[**Supplementary Table 3. Comparisons of outcomes between E+MS and MS.** 7](#_Toc136030744)

[**Supplementary Table 4. Comparisons of outcomes between E+MS (hybrid and multi-staged operation) and MS.** 8](#_Toc136030745)

**Supplementary Method 1. Protocol for data quality management.**

1. Definition of variables were discussed and unified according to the terminology reporting standards or published paper before the initiation of data collection. Clinical research coordinators (CRCs) and neurosurgery residents were then trained by cerebrovascular neurosurgeons with more than 15 years’ working experience. CRCs were responsible for demographic information and follow-up data, and neurosurgery residents for angiographic features. The two parts were blinded to each other to ensure the data collected were not biased by imaging characteristics or clinical outcomes.
2. A standard training dataset with 50 cases were used to check the consistency of data collectors. For those variables or cases with significant interobserver variation, the consensus was reached by either modifying the confusing definitions or retraining the data collectors. Only when the consistency reached 90% can the CRC or the resident allowed to extracting information independently.
3. While recording data, one could ask for help about unsure cases in a discussion group with cerebrovascular neurosurgeons in it, or mark these cases and discuss in weekly meetings.
4. The group leader with more than five years’ working experience randomly spot checks these data biweekly. Investigators would receive training again if their data were of low quality, and these data would be recollected by other investigators.

**Supplementary Table 1. Number and proportional (%) breakdown of characteristics in patients lost to follow-up.**

|  | Patients, No. (%) | | |  |
| --- | --- | --- | --- | --- |
| Characteristics | CM | MS | E+MS | P |
| Sample size | 10 | 74 | 9 |  |
| Female | 4 (40.0) | 22 (29.7) | 4 (44.4) | 0.205 |
| Age at diagnosis (median (IQR)) | 40.5 (21.8–49.3) | 25.2 (18.9–33.6) | 20.8 (16.2–30.1) | 0.570 |
| mRS at admission |  |  |  | - |
| 0 | 4 (40.0) | 10 (13.5) | 0 (0.0) |  |
| 1 | 5 (50.0) | 57 (77.0) | 9 (100.0) |  |
| 2 | 1 (10.0) | 7 (9.5) | 0 (0.0) |  |
| 3 | 0 (0.0) | 0 (0.0) | 0 (0.0) |  |
| Seizure | 1 (10.0) | 40 (54.1) | 8 (88.9) | 0.002 |
| Headache | 3 (30.0) | 30 (40.5) | 1 (11.1) | 0.201 |
| Neurological deficit | 2 (20.0) | 10 (13.5) | 1 (11.1) | 0.828 |
| Location |  |  |  |  |
| Frontal | 4 (40.0) | 29 (39.2) | 1 (11.1) | 0.248 |
| Temporal | 2 (20.0) | 29 (39.2) | 1 (11.1) | 0.147 |
| Parietal | 4 (40.0) | 14 (18.9) | 5 (55.6) | 0.027 |
| Occipital | 0 (0.0) | 17 (23.0) | 4 (44.4) | 0.068 |
| Cerebellum | 1 (10.0) | 2 (2.7) | 0 (0.0) | 0.400 |
| Basal ganglia | 0 (0.0) | 0 (0.0) | 0 (0.0) | - |
| Spetzler-Martin grade |  |  |  | 0.149 |
| 1 | 1 (10.0) | 10 (13.5) | 0 (0.0) |  |
| 2 | 5 (50.0) | 22 (29.7) | 3 (33.3) |  |
| 3 | 1 (10.0) | 36 (48.6) | 5 (55.6) |  |
| 4 | 3 (30.0) | 6 (8.1) | 1 (11.1) |  |
| Ventricular system involvement | 2 (20.0) | 14 (18.9) | 2 (22.2) | 0.971 |
| Size |  |  |  | 0.928 |
| <3cm | 3 (30.0) | 20 (27.0) | 2 (22.2) |  |
| ≥3cm | 7 (70.0) | 54 (73.0) | 7 (77.8) |  |
| Eloquent region | 5 (50.0) | 34 (45.9) | 5 (55.6) | 0.848 |
| Feeding artery dilation | 7 (70.0) | 47 (63.5) | 6 (66.7) | 0.913 |
| Single feeder | 0 (0.0) | 13 (17.6) | 2 (22.2) | 0.319 |
| Perforating artery | 2 (20.0) | 11 (14.9) | 1 (11.1) | 0.860 |
| Aneurysm | 0 (0.0) | 9 (12.2) | 1 (11.1) | 0.507 |
| Diffuse nidus | 2 (20.0) | 12 (16.2) | 3 (33.3) | 0.450 |
| Any deep drainage | 4 (40.0) | 17 (23.0) | 2 (22.2) | 0.495 |
| Draining vein stenosis | 2 (20.0) | 9 (12.2) | 0 (0.0) | 0.395 |
| Venous aneurysm | 8 (80.0) | 29 (39.2) | 3 (33.3) | 0.041 |

**Supplementary Figure 1. Overlap assessment**


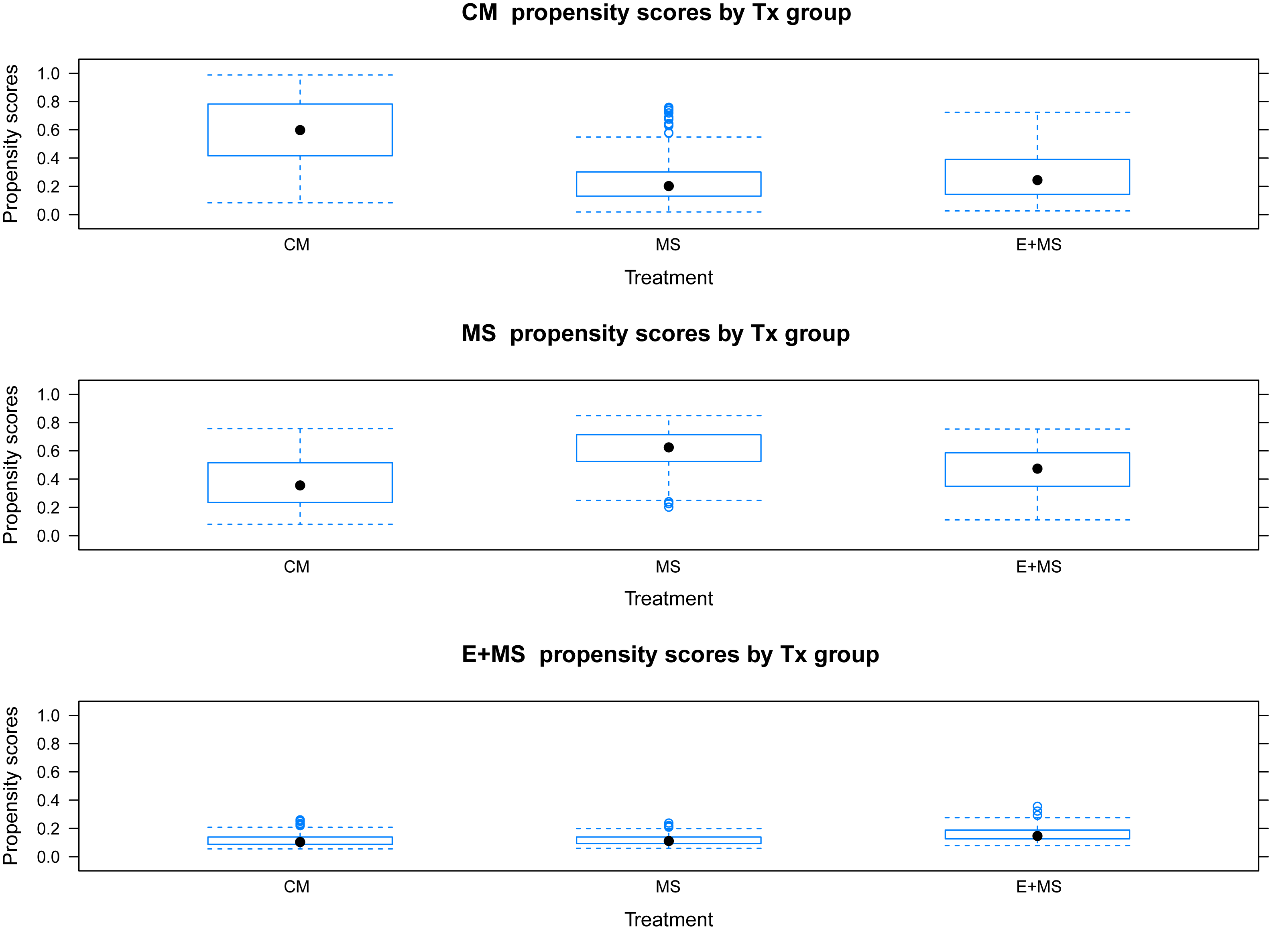


Each panel presents box plots by treatment group of the estimated propensity scores for one of the treatments. CM: conservative management; MS: microsurgery only; E+MS: microsurgery with preoperative embolization.

**Supplementary Figure 2. Effect size plots for assessing the balance of pretreatment variables**


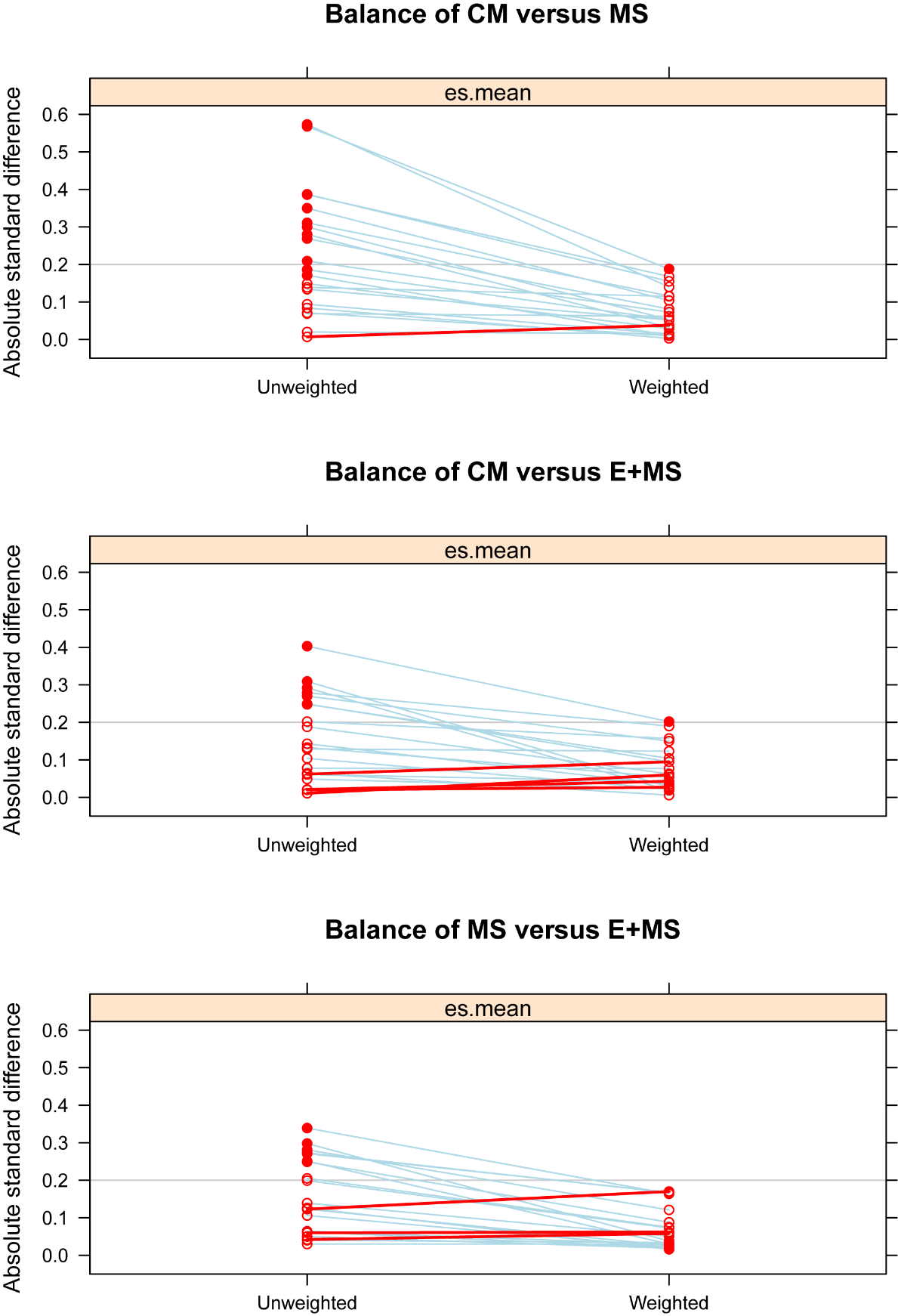


CM: conservative management; MS: microsurgery only; E+MS: microsurgery with preoperative embolization.

**Supplementary Table 2.** **Hazard ratios of different therapeutic strategies after 3, 5, and 10 years for symptomatic hemorrhagic stroke and death.**

| Comparison | First 3 years | First 5 years | First 10 years |
| --- | --- | --- | --- |
| MS vs CM | 5.58 (0.62–50.29) | 0.43 (0.14–1.30) | 0.21 (0.07–0.60) |
| E+MS vs CM | 21.80 (2.50–190.00) | 1.33 (0.40–4.46) | 1.51 (0.53–4.38) |
| E+MS vs MS | 4.19 (1.24–14.11) | 3.79 (1.09–13.14) | 4.69 (1.35–16.30) |

Values are expressed as hazard ratio with 95% confidence interval.

CM: conservative management; MS: microsurgery only; E+MS: microsurgery with preoperative embolization.

**Supplementary Table 3. Comparisons of outcomes between E+MS and MS.**

|  | OR | 95% CI | P |
| --- | --- | --- | --- |
| **Primary outcome** |  |  |  |
| mRS >2 | 1.37 | 0.56 – 3.34 | 0.494 |
| Worsening mRS | 2.02 | 1.08 – 3.77 | 0.027 |
| Symptomatic hemorrhagic stroke and death* | 4.76 | 1.37 – 16.55 | 0.014 |
| **Secondary outcome** |  |  |  |
| Increased frequency of seizures | 1.82 | 0.37 – 8.83 | 0.460 |
| New-onset transient ND | 1.82 | 0.99 – 3.36 | 0.054 |
| New-onset permanent ND | 2.53 | 0.90 – 7.10 | 0.079 |

*The effect size was measured by hazard ratio.

CI: confidence interval; E+MS: microsurgery with preoperative embolization; mRS: modified Rankin scale; MS: microsurgery only; ND: neurological deficit; OR: odds ratio.

**Supplementary Table 4. Comparisons of outcomes between E+MS (hybrid and multi-staged operation) and MS.**

|  | MS | |  | Single-staged hybrid operation | | | |  | Multi-staged operation | | | |
| --- | --- | --- | --- | --- | --- | --- | --- | --- | --- | --- | --- | --- |
|  | Event | Event rate (%) |  | Event | Event rate (%) | OR (95% CI) | P |  | Event | Event rate (%) | OR (95% CI) | P |
| **Primary outcome** |  |  |  |  |  |  |  |  |  |  |  |  |
| mRS >2 | 25.2 | 7.9 |  | 4.1 | 8.9 | 1.14 (0.38–3.40) | 0.813 |  | 2.7 | 14.3 | 1.96 (0.50–7.62) | 0.331 |
| Worsening mRS | 50.1 | 15.6 |  | 12.4 | 26.7 | 1.97 (0.96–4.03) | 0.064 |  | 5.3 | 28.4 | 2.15 (0.75–6.13) | 0.153 |
| Symptomatic hemorrhagic stroke and death* | 4.2 | 0.22 |  | 0.0 | 0.0 | - | - |  | 3.9 | 3.61 | 18.13 (5.22–62.92) | <0.001 |
| **Secondary outcome** |  |  |  |  |  |  |  |  |  |  |  |  |
| Increased frequency of seizures | 5.9 | 1.9 |  | 2.2 | 4.6 | 2.58 (0.53–12.66) | 0.243 |  | 0.0 | 0.0 | - | - |
| New-onset transient ND | 56.9 | 17.7 |  | 13.7 | 29.4 | 1.93 (0.97–3.87) | 0.062 |  | 4.7 | 25.2 | 1.57 (0.53–4.62) | 0.417 |
| New-onset permanent ND | 11.9 | 3.7 |  | 1.5 | 3.1 | 0.84 (0.15–4.85) | 0.850 |  | 4.3 | 23.0 | 7.78 (2.29–26.47) | 0.001 |

*The event rate was reported as incidence rate per 100 person-years and the effect size was measured by hazard ratio.

CI: confidence interval; CM: conservative management; E+MS: microsurgery with preoperative embolization; mRS: modified Rankin scale; MS: microsurgery only; ND: neurological deficit; OR: odds ratio.
